# Multidrug- and Extensively Drug-Resistant *Mycobacterium tuberculosis* Beijing Clades, Ukraine, 2015

**DOI:** 10.3201/eid2603.190525

**Published:** 2020-03

**Authors:** Matthias Merker, Elena Nikolaevskaya, Thomas A. Kohl, Barbara Molina-Moya, Olha Pavlovska, Patrik Brännberg, Andrii Dudnyk, Valentyna Stokich, Ivan Barilar, Iryna Marynova, Tetiana Filipova, Cristina Prat, Anders Sjöstedt, Jose Dominguez, Olena Rzhepishevska, Stefan Niemann

**Affiliations:** Research Center Borstel, Borstel, Germany (M. Merker, T.A. Kohl, I. Barilar, S. Niemann);; Odessa Regional TB Hospital, Odessa, Ukraine (E. Nikolaevskaya, O. Pavlovska, V. Stokich);; Institut d'Investigació Germans Trias i Pujol, Badalona, Spain (B. Molina-Moya, C. Prat, J. Dominguez);; Centro de Investigación Biomédica en Red (CIBERES), Badalona (B. Molina-Moya, C. Prat, J. Dominguez);; Universitat Autònoma de Barcelona, Barcelona, Spain (B. Molina-Moya, C. Prat, J. Dominguez);; Umeå University, Umea, Sweden (P. Brännberg, A. Sjöstedt, O. Rzhepishevska);; National Pirogov Memorial Medical University, Vinnytsia, Ukraine (A. Dudnyk);; Odessa I.I. Mechnikov National University, Odessa (I. Marynova, T. Filipova);; German Center for Infection Research, Hamburg-Lübeck-Borstel-Riems, Germany (S. Niemann)

**Keywords:** *Mycobacterium tuberculosis*, tuberculosis, drug resistance, whole-genome sequencing, contact tracing, MDR TB, XDR TB, antimicrobial resistance, Ukraine, Beijing lineage, tuberculosis and other mycobacteria

## Abstract

Multidrug-resistant (MDR) and extensively drug-resistant (XDR) tuberculosis (TB) is an emerging threat to TB control in Ukraine, a country with the third highest XDR TB burden globally. We used whole-genome sequencing of a convenience sample to identify bacterial genetic and patient-related factors associated with MDR/XDR TB in this country. MDR/XDR TB was associated with 3 distinct *Mycobacterium tuberculosis* complex lineage 2 (Beijing) clades, Europe/Russia W148 outbreak, Central Asia outbreak, and Ukraine outbreak, which comprised 68.9% of all MDR/XDR TB strains from southern Ukraine. MDR/XDR TB was also associated with previous treatment for TB and urban residence. The circulation of Beijing outbreak strains harboring broad drug resistance, coupled with constraints in drug supply and limited availability of phenotypic drug susceptibility testing, needs to be considered when new TB management strategies are implemented in Ukraine.

Each year, approximately half a million new cases of multidrug-resistant (MDR) tuberculosis (TB) challenge global health (*1**,**2*). MDR TB is caused by *Mycobacterium tuberculosis* complex (MTBC) strains, resistant to at least isoniazid and rifampin (*3*). In Europe, Ukraine is a hotspot of drug-resistant TB, with 6,564 laboratory-confirmed MDR and rifampin-resistant cases (*2*) and the third highest burden of extensively drug-resistant (XDR) TB (1,097 laboratory confirmed cases) globally in 2017 (*2*). XDR TB is a complicated form of MDR TB with additional resistances to >1 second-line injectable antimicrobial drug and a fluoroquinolone (*1*). Treatment of XDR TB can take up to 2 years (*4*), and treatment of a single XDR TB case has been reported to exceed €100,000 (*5**,**6*), even though treatment success rates remain ≈30% in the European Region of the World Health Organization (40 countries reported) (*7*). Improvement of MDR/XDR TB prevention, diagnosis, and treatment is one of the core activities prioritized by WHO and the European Respiratory Society to eliminate TB (*6*).

In addition to shortcomings in TB diagnosis and treatment, bacterial genetic factors might play a role in the epidemiologic success of certain MDR strains, especially of lineage 2 (Beijing) in Eurasia (*8**–**11*). Beijing MDR outbreak strains were shown to acquire fitness-enhancing mutations (i.e., mutations that increase in vitro growth rates) that may result in higher virulence and increased transmissibility, thus fostering the MDR TB epidemic in Eastern Europe (*8**–**10*). In line with this assumption, recent computational models predict that in many high TB incidence countries, person-to-person transmission but not treatment-related acquisition accounts for almost all (95.9%) incident MDR TB cases (*12*). Whole-genome sequencing (WGS) coupled with a molecular drug resistance prediction has provided insight into MTBC transmission networks and the transmissibility of MDR/XDR MTBC strains (*8**–**10**,**13**–**15*). We applied a WGS-based molecular epidemiologic approach to identify molecular resistance patterns, dominant strain types, and patient-related factors associated with MDR/XDR TB in a convenience sample from southern Ukraine.

## Methods

### Study Population

MTBC isolates were collected during January–June 2015 in the clinical laboratory of Odessa Regional TB Hospital (now Odessa Regional Center for Socially Significant Diseases; Odessa, Ukraine), where most clinical MTBC cultures from the region are routinely isolated and analyzed. We completed subculturing and WGS on a subset of culture isolates obtained at the clinical laboratory during the study period. The sampling strategy aimed to include a similar proportion of MDR/XDR TB isolates and non-MDR (polyresistant and pansusceptible) strains. We included samples on the basis of routine phenotypic rifampin and isoniazid susceptibility testing that categorized isolates as MDR or non-MDR TB strains; otherwise, inclusion was unbiased and isolates were included directly from the clinical pipeline when human and technical resources were available. Isolates from both new and retreatment cases were eligible for inclusion; retreatment cases included relapse, reinfection, failure of treatment, or interrupted treatment episode. In accordance with the clinical protocol in the laboratory, all MTBC cultures that were not included in our study were autoclaved and disposed.

**Figure 1:**
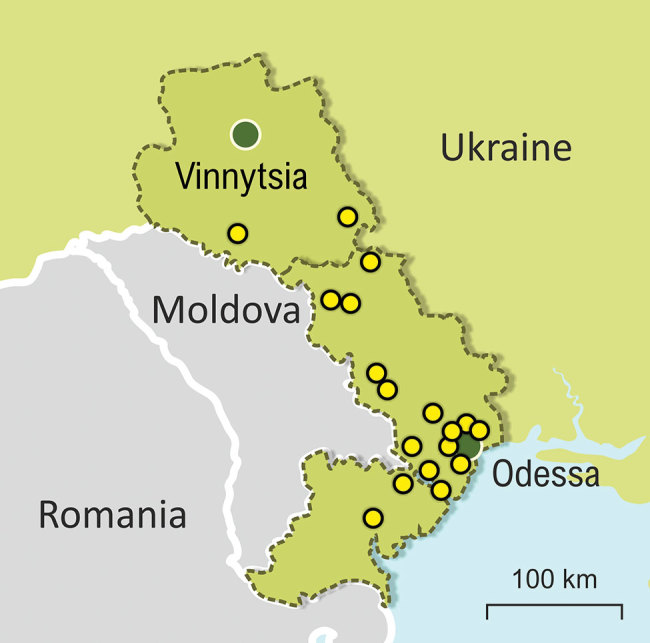
Locations where *Mycobacterium tuberculosis* complex (MTBC) DNA samples were collected in Odessa and Vinnytsia regions. Yellow dots indicate locations of patients infected with a MTBC lineage 2 Ukraine outbreak strain. Green dots indicate major cities.s

Our study was mainly set in the Odessa region, which had the highest TB burden (3,039 cases) in Ukraine, the second highest MDR TB burden (30.5% for new cases and 46.9% for retreatment), and TB incidence twice as high as the country’s average (127.9 cases/100,000 population) in 2017 (*16*). In total, we included 186 MTBC isolates, each obtained from 1 patient; most were living in the Odessa region (95.2%). The rest of the samples (4.8%) were from the bordering administrative region of Vinnytsia (Figure 1). Inclusion of these samples supported investigation of epidemiologic links between both regions. Thus, the analysis is focused on the differences between 103 non-MDR and 74 MDR/XDR MTBC strains all isolated from southern Ukraine. Patient characteristics assessed for association with MDR/XDR TB were age, sex, residence, HIV status, and case definition.

### Contact Tracing of Patients Infected with a Ukraine Outbreak Strain

We conducted a retrospective review of epidemiologic contact tracing for patients infected with a Ukraine outbreak strain through patient records. Because Ukraine lacks resources for contact tracing and TB is stigmatized, we anticipated that the journal information would yield few contacts. To assist with contact identification, we interviewed physicians providing care for the patients with a Ukraine outbreak strain of TB. We maintained data confidentiality in accordance with regional ethics approvals in Ukraine and Umea, Sweden (N 2012-292-31M). We immediately shared information regarding nosocomial transmission of Ukraine outbreak strains with the administration of Odessa Regional TB Hospital to support infection control measures.

### Phenotypic Characterization of Drug Resistance and DNA Isolation

We performed primary isolation and drug-susceptibility testing of MTBC strains using clinical diagnostics protocol for TB in Ukraine. In brief, we treated sputum samples with the N-acetyl-L-cysteine-NaOH (NALC) method for decontamination and further processed them for Ziehl-Neelsen staining using GeneXpert-MTB/RIF (Cepheid, https://www.cepheid.com), followed by inoculation on Löwenstein–Jensen (LJ) medium and BACTEC MGIT960 tubes (Becton Dickinson, https://www.bd.com) for culture confirmation and subsequently for phenotypic drug susceptibility testing. We used the following drug concentrations (mg/L, by medium): isoniazid, LJ 0.2, MGIT960 0.1; rifampin, LJ 40.0, MGIT960 1.0; ethambutol, LJ 2.0, MGIT960 5.0; pyrazinamide, MGIT960 100.0; and streptomycin, LJ 4.0, MGIT960 1.0. Individual reagents were not always available for routine drug-susceptibility testing, and resistance profiles in this manuscript are based on molecular markers (Appendix 1 Table 1, https://wwwnc.cdc.gov/EID/article/26/3/19-0525-App1.xlsx). We extracted mycobacterial DNA from LJ cultures as previously described (*17*) using cetyltrimethylammonium bromide.

### Whole-Genome Sequencing

We completed WGS using Illumina technology (MiSeq, NextSeq 500, and Nextera XT library preparation kit; Illumina, https://www.illumina.com) according to the manufacturer’s instructions. We mapped raw read data (FASTQ files) to the *M. tuberculosis* H37Rv genome (GenBank accession no. NC_000962.3) using Burrows-Wheeler Aligner maximal exact matches (*18*) and refined mappings with the Genome Analysis Toolkit software package (*19*). We detected variants, including single-nucleotide polymorphisms (SNPs) and insertions and deletions (indels), with Samtools mpileup (*20*). For a concatenated sequence alignment, the basis for the phylogenetic reconstruction, we considered only SNPs with minimum thresholds of 4 reads in both forward and reverse orientation, 4 reads calling the SNP with a Phred score >20, and 75% SNP frequency. We excluded consecutive SNPs for the phylogenetic reconstruction detected within <12 bp, which can occur as artificial variants around indels and which would inflate the genetic distance of individual isolates. We combined all remaining SNP positions that had a clear base call for all strains and matched the threshold levels in >95% of all strains in one FASTA alignment, further excluding repetitive regions and resistance-associated genes.

### Phylogenetic Reconstruction

We calculated maximum-likelihood trees with FastTree version 2.1.9 (*21*) using a general time-reversible nucleotide substitution model and 1,000 resamplings. The consensus tree was rooted with the midpoint root option in FigTree version 1.4 (http://tree.bio.ed.ac.uk/software/figtree). We obtained cladograms of outbreak strains and number of branch-specific mutations by maximum parsimony using BioNumerics version 7.6 (Applied Maths, https://www.applied-maths.com).

We inferred phylogenetic lineages from specific SNPs based on Coll et al. (*22*) and Merker et al. (*8*). As proxy for TB cases associated with direct transmission events, we considered a maximum pairwise genetic distance between >2 MTBC isolates of 5 SNPs, proposed by Walker et al. as a strict threshold that identified cases associated with household transmission (*23*).

### Genotypic Drug Resistance Prediction

We extracted polymorphisms from 37 drug resistance– and bacterial fitness–associated genomic targets (Appendix 1 Table 1). We excluded known and newly identified phylogenetic, non–resistance-related variants (*8**,**24**,**25*) from the genotypic drug resistance prediction (Appendix 1 Table 3).

Resistance genotypes were defined as follows: for wild-type alleles (H37Rv reference sequence) for all resistance-associated targets we inferred antimicrobial susceptibility (genotypic wild type, gWT). We classified isolates with unknown mutations as genotypic non–wild-type (non-WT) with no further classification. We considered isolates with known resistance-mediating mutations to be resistant to the respective antimicrobial drug (Appendix 1 Table 1). Using genotypic resistance predictions, we classified the isolates as XDR (known mutations mediating resistance to isoniazid, rifampin, >1 second-line injectable drug, and a fluoroquinolone), MDR (isoniazid and rifampin resistance but not XDR), and non-MDR (either pansusceptible or any resistance but not MDR).

### Statistical Analysis

We created an association plot in R version 3.3 (https://www.r-project.org) using the vcd package version 1.4. The underlying assumption is that the proportion of MDR/XDR MTBC strains within 1 group resembles the proportion of this group among all analyzed isolates (i.e., the resistance level is independent from the phylogenetic groupings). We calculated Pearson residuals to measure the departure from independence from each cell; values of +2 represent significant deviation at α = 0.05 and values of +4 deviation at α = 0.001 (*26*). We prepared a box plot of pairwise genomic distances of MTBC strains from defined strain groups with R version 3.3 using the ggplot2 package version 2.2 (Appendix 2 Figure 1).

We analyzed factors associated with MDR/XDR TB by logistic regression (univariate and multivariate model) using the glm function in R version 3.3.1 (*27*). We excluded the variable MTBC genotype from multivariate analysis because cell counts included 0. We compared means of pairwise SNP distances with 1-way analysis of variance (ANOVA) and Bonferroni multiple comparison tests.

## Results

### Study Population

During the study period, TB service identified 1,026 patients with >1 culture–positive specimen in the Odessa region. Of these cultures, 330 isolates were identified as MDR.

Our study included 16.17% (169/1,026) of the identified positive cultures. MDR/XDR TB specimens constituted 21.82% (72/330) of all MDR/XDR TB–positive cultures identified during the study period. The remaining 97 isolates were phenotypically non-MDR MTBC strains (50 pansusceptible and 47 monoresistant or polyresistant strains). In addition, we randomly collected and analyzed 6 pansusceptible and 2 MDR/XDR MTBC isolates from TB patients registered in the bordering region of Vinnytsia (Vinnytsia Regional TB Hospital, Vinnytsia).

After completing WGS, we withdrew 4.8% (9/186) of MTBC isolates from analysis because of discrepancies between phenotypic drug susceptibility testing and the applied genome-based drug resistance prediction. Therefore, we included 177 MTBC clinical isolates in analysis.

### MDR/XDR MTBC Beijing Outbreak Clades

**Figure 2:**
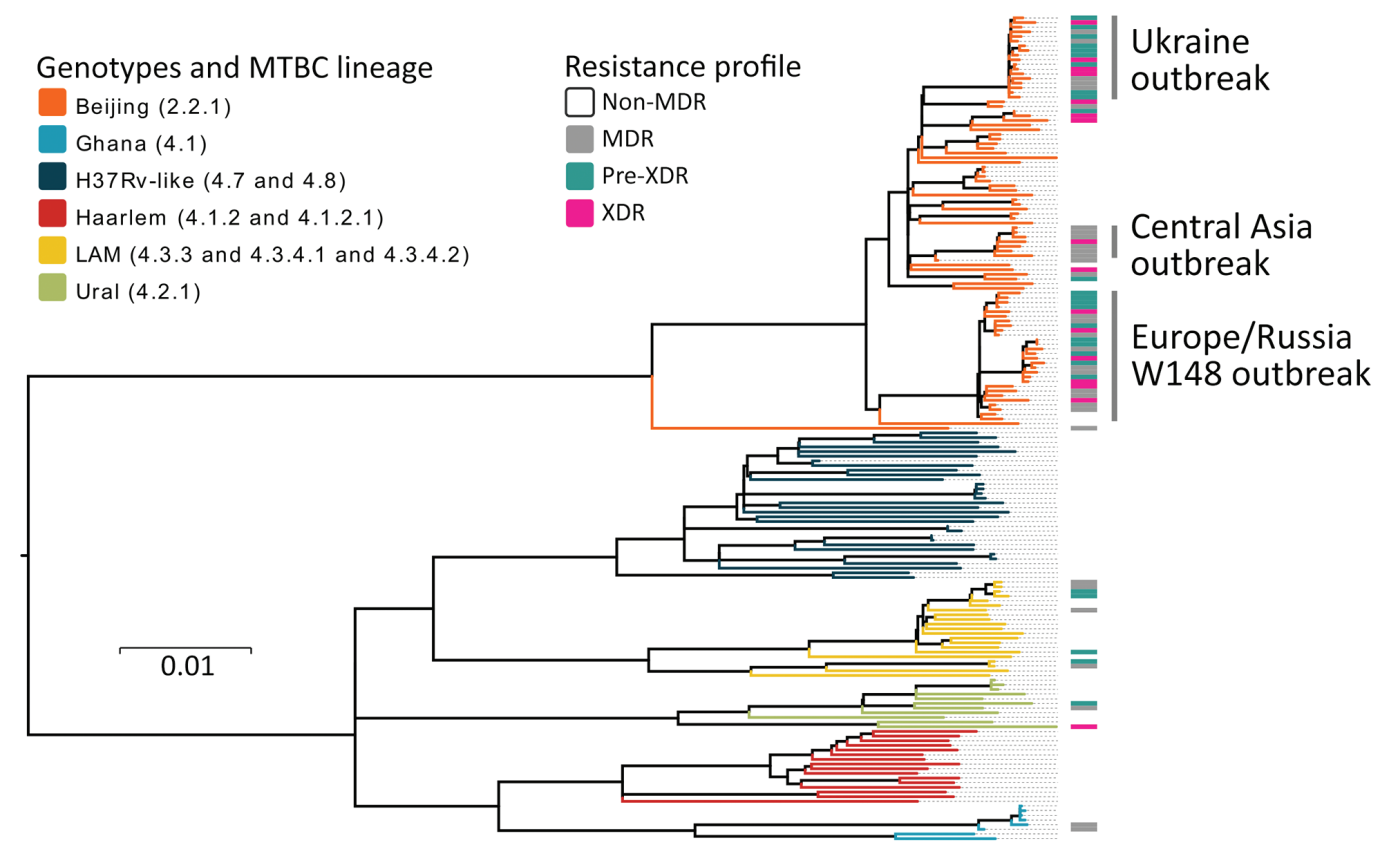
Maximum-likelihood phylogeny based on 10,339 SNPs, and employing general time-reversible substitution model for 177 clinical MDR/XDR and non-MDR *Mycobacterium tuberculosis* complex isolates from southern Ukraine. Branches are color-coded according to the phylogenetic classification from Coll et al. (*22*). Resistance profile bars represent drug resistance classifications based on drug resistance mediating mutations. Scale bar indicates substitutions per site. MDR, multidrug resistant; XDR, extensively drug-resistant.

We classified a total of 177 clinical isolates based on WGS data from 10,339 variable positions, according to a recently proposed SNP barcode (*22*). Furthermore, we classified 89 of the isolates (50.3%) as Beijing genotype (lineage 2.2.1) and the remaining 88 (49.7%) as Euro-American lineage 4. We differentiated lineage 4 strains to the following genotypes and sublineages: 8/88 (9.1%) Ghana (lineage 4.1); 32/88 (36.4%) H37Rv-like (lineages 4.7 and 4.8); 16/88 (18.2%) Haarlem (lineages 4.1.2 and 4.1.2.1); 21/88 (23.9%) LAM (lineages 4.3.3, 4.3.4.1, and 4.3.4.2); and 11/88 (12.5%) Ural (lineage 4.2.1) (Figure 2).

Within Beijing lineage 2.2.1, we identified 3 closely related subgroups that exhibited a lower genetic diversity compared with other lineage 2.2.1 strains, based on intragroup pairwise SNP distances between any 2 strains. Two of those subgroups were previously reported in other settings (*8*); we identified them by clade-specific SNPs, and termed them Central Asia outbreak and Europe/Russia W148 outbreak. The third subgroup, named Ukraine outbreak, had not been previously described. These Beijing clades showed a lower median pairwise SNP distance of 25 (interquartile range [IQR] 20.0–32.5) for Central Asia outbreak, 39 (IQR 25.0–48.0) for Europe/Russia W148 outbreak, and 14 (IQR 11.5–18.0) for Ukraine outbreak, contrasting with a distance of 122 (IQR 170.8–139.0) for other Beijing strains (p<0.05, one-way ANOVA, Bonferroni multiple comparison test) (Appendix 2 Figure 1).

When we considered a strict threshold of 0–5 SNPs, as previously proposed for MTBC strains isolated from household contacts (*23*), only 9.0% of all patients (16/177) could be linked to 7 putative transmission networks comprising 2–4 patients each. We found these networks or molecular clusters among the 3 Beijing outbreaks (3 transmission events) and other Beijing strains (1 transmission event), and also among Euro-American strains (lineages 4.1 and 4.8; 3 transmission events) (Appendix 1 Table 1).

### Contact Tracing Review for Beijing Ukraine Outbreak Cases

Epidemiologic links between clustered patients were partially confirmed by retrospective contact tracing review of patients infected with the Ukraine outbreak strain; of 18 cases, 7 were new and 11 retreatment (Figure 1; Appendix 1 Table 4; Appendix 2 Figure 2). Ukraine outbreak cases were registered in several places (Figure 1); 3 patients had contact through a psychiatric hospital (patient isolate odir-1606, with pairwise SNP distance of 8; odir-1746, SNP distance 10; odir-1747, with distance of 12) and 2 cases (odir-5192 and odir-1636, SNP distance 3) had close contact through their immediate neighborhood (Appendix 1 Table 4; Appendix 2 Figure 2). The neighborhood contact had also been detected by applying the strict SNP threshold. Three other case-patients had a close family MDR TB contact, but bacterial DNA was not available for these cases. No apparent connection could be established among 13 of 18 Ukraine outbreak cases.

### Patient Factors Associated with MDR/XDR TB

To pinpoint certain demographic and treatment-related factors associated to MDR/XDR TB in southern Ukraine, we further used logistic regression analysis (Table 1). After multivariate analysis, 3 factors—previous TB treatment (OR 3.5, 95% CI 1.0–12.8; p = 0.04), living in a city (OR 6.0, 95% CI not applicable; p = 0.005), and infection with a Beijing outbreak strain (OR 7.4, 95% CI 1.8–29.9; p<0.001) —were significantly associated with MDR/XDR TB. Multivariate analysis included the additional variables age, gender, HIV status, and MTBC genotype.

**Table 1:** Factors associated with MDR/XDR TB in southern Ukraine, analyzed by logistic regression*

Factor	No. (%) cases MDR/XDR TB, n = 74	Univariate analysis			Multivariate analysis†	
		OR (95% CI)	p value		Adjusted OR (95% CI)	Adjusted p value
Age, y						
<30	15 (20.3)	**3.0 (1.0–8.3)**	**0.04**		0.9 (0.2–4.0)	0.87
30–39	23 (31.1)	1.5 (0.6–3.5)	0.39		0.9 (0.3–3.1)	0.95
40–49	24 (32.4)	1.6 (0.7–3.9)	0.27		0.8 (0.1–5.2)	0.78
>50	12 (16.2)	Referent				
Case						
Previous treatment	33 (44.6)	**3.3 (1.7–6.5)**	**<0.001**		**3.5 (1.0–12.8)**	**0.04**
New case	41 (55.4)	Referent				
Sex						
M	50 (67.6)	0.9 (0.5–1.8)	0.85		3.0 (0.6–15.5)	0.12
F	24 (32.4)	Referent				
HIV status						
Positive	24 (32.4)	1.5 (0.8–2.9)	0.23		0.8 (0.1–6.2)	0.81
Negative	50 (67.6)	Referent				
Residence						
Unknown	5 (6.8)	0.5 (0.1–1.5)	0.21		0.7 (NA)†	0.68
City	27 (36.5)	**2.1** **(1.1–4.2)**	**0.03**		**6.0 (NA)†**	**0.005**
Village	42 (56.8)	Referent				
Outbreak						
Yes	51 (68.9)	**112** **(25.4–493.4)**	**≤0.001**		**7.4 (1.8–29.9)**	**≤0.001**
No	23 (31.1)	Referent				
MTBC genotype†						
Beijing	61 (82.4)	**3.5 (1.3–9.5)**	**0.01**			
H37Rv-like	0 (0)	NA	0.99			
Ghana	2 (2.7)	0.5 (0.1–3.4)	0.51			
Haarlem	0(0)	NA	0.99			
Ural	3 (4.1)	0.6 (0.1–3.0)	0.54			
LAM	8 (10.8)	Referent				

*Bold text indicates significance (p≤0.05). MDR, multidrug-resistant; MTBC, *Mycobacterium tuberculosis* complex; NA, not available; OR, odds ratio; TB, tuberculosis; XDR, extensively drug resistant. †MTBC genotype contains categories with 0 events; thus, in the multivariate analysis, OR could not be calculated and OR 95% CI reached infinity.

### Transmitted and Acquired Drug Resistance among Beijing Outbreak Strains

**Figure 3:**
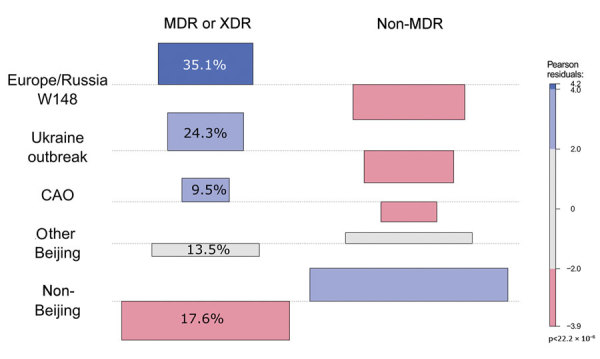
Association plot comparing expected and observed numbers of MDR/XDR and non-MDR strains from different phylogenetic groups. The colors and bar heights reflect the Pearson residuals, the width of the boxes is proportional to the square root of the expected cell counts, blue squares reflect values that are overrepresented, and red squares reflect values that are underrepresented. Pearson values of +2 represent significant deviation at α = 0.05 level, and values of +4 represent significant deviation at α = 0.001 level. For example, there are more MDR/XDR TB strains in the Europe/Russia W148 clade (35.1% of all MDR/XDR strains) than expected under the hypothesis of independence. CAO, Central Asian outbreak; MDR, multidrug resistant; XDR, extensively drug-resistant.

Consistent with results from the phylogenetic and logistic regression analyse, Beijing outbreak strains were the main carriers of MDR/XDR TB. The Europe/Russia W148, Central Asia, and Ukraine outbreak strains accounted for 29.9% of the cohort, but they contributed to more than two thirds (68.9%) of all MDR/XDR TB cases (Figure 3; Appendix 1 Table 2). The 3 Beijing outbreak strains all harbored high rates of first-line drug resistances to isoniazid (93%–100%), rifampin (93%–100%), streptomycin (93%–100%), ethambutol (86%–100%), and pyrazinamide (75%–100%) (Table 2). In contrast, first-line drug resistance rates among all other Beijing and non-Beijing strains were reduced (8%–33% for individual drugs). Similarly, resistance rates to second-line drugs among the 3 outbreak strains were high (ofloxacin 14%–44%, kanamycin 14%–50%, and prothionamide 57%–100%) compared with other Beijing (ofloxacin 11%, kanamycin 17%, and prothionamide 11%) and non-Beijing (ofloxacin 3%, kanamycin 8%, and prothionamide 15%) strains (Table 2).

**Table 2:** Percentages of resistance to antimicrobial drugs among *Mycobacterium tuberculosis* strains identified in southern Ukraine*

Drug	All strains, n = 177	Ukraine outbreak, n = 18	Europe/Russia W148 outbreak, n = 28	Central Asia outbreak, n = 7	Other Beijing strains, n = 36	Non-Beijing strains, n = 88
Isoniazid	**48.0** (54.2)	**100** (100)	**92.9** (92.9)	**100** (100)	**27.8** (44.4)	**27.3** (33.0)
Rifampin	**42.9** (44.3†)	**100** (100)	**92.9** (92.9)	**100** (100)	**30.6** (31.4†)	**15.9** (18.2)
Streptomycin	**45.2** (54.8)	**100** (100)	**92.9** (92.9)	**100** (85.7)	**33.33** (47.2)	**19.32** **(**34.1)
Ethambutol	**37.3** (36.2)	**100** (66.7)	**85.7** (75.0)	**100** (57.1)	**11.11** (38.9)	**14.77** (14.8)
Pyrazinamide	**31.6**	**88.9**	**75**	**100**	**13.9**	**8.0**
Ofloxacin	**14.7**	**44.4**	**35.7**	**14.3**	**11.1**	**3.4**
Amikacin	**9.6**	**22.22**	**21.43**	**14.3**	**8.3**	**3.4**
Capreomycin	**9.6**	**22.2**	**21.4**	**14.3**	**8.3**	**3.4**
Kanamycin	**19.2**	**50.0**	**39.3**	**14.3**	**16.7**	**8.0**
Prothionamide	**34.5**	**100**	**78.6**	**57.1**	**11.1**	**14.8**
Cycloserine	**0**	**0**	**0**	**0**	**0**	**0**
Linezolid	**1.0**	**0**	**0**	**0**	**0**	**1.1**
PAS	**10.7**	**0**	**39.3**	**0**	**0**	**9.**1

*Percentages of genotypic (parentheses) and phenotypic (bold text) drug resistance for *Mycobacterium tuberculosis* complex strains in southern Ukraine. PAS, para-aminosalicylic acid. †One phenotypic susceptibility test result was not available.

An association plot confirmed the results from the logistic regression analysis that demonstrated that MDR/XDR TB among patients in southern Ukraine is clearly linked to the 3 Beijing outbreak clades and that drug resistance is not equally distributed among the MTBC strains, as one would expect from random treatment failures (i.e., acquired resistance) (Figure 3). For example, the Ukraine outbreak strain, constituting 10.2% of the total cohort, was associated with 24.3% of all MDR/XDR TB cases. In contrast, non-Beijing strains, constituting 49.7% of the cohort, were associated with only 17.6% of all MDR/XDR cases.

**Figure 4:**
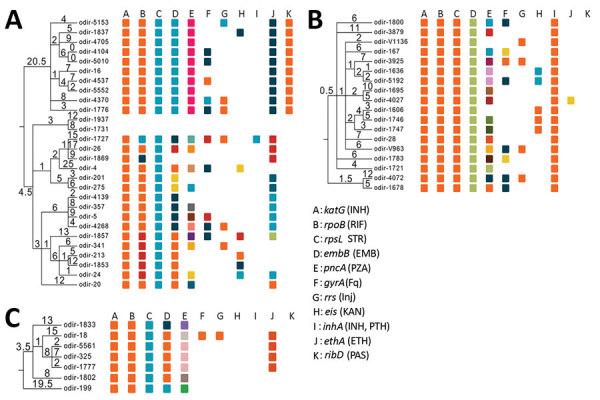
Maximum parsimony phylogenies of 3 *Mycobacterium tuberculosis* complex Beijing clades in Ukraine: Europe/Russia W148 outbreak (A), Ukraine outbreak (B), and Central Asian outbreak (C). Mutations mediating drug resistance to different antibiotics are color coded. Same color indicates identical mutation. Numbers on branches indicate the number of unique mutations. EMB, ethambutol; ETH, ethionamide; Fq, fluoroquinolones; INH, isoniazid; Inj, second-line injectable drugs (amikacin, kanamycin, capreomycin); KAN, kanamycin; PAS, para-aminosalicylic acid; PTH, prothionamide; PZA, pyrazinamide; RIF, rifampin; STR, streptomycin.

Strains from the 3 outbreaks demonstrated clade-specific drug resistance–related mutations, mainly to first-line drugs (Figure 4). All Ukraine outbreak strains shared identical mutations mediating resistance to isoniazid (*katG* S315T, *inhA* −15c/t), prothionamide (*inhA* −15c/t), rifampin (*rpoB* S450L), streptomycin (*rpsL* K88R), and ethambutol (*embA* −12 c/t and *embB* Y334H), suggesting that patients have been infected primarily with a strain already resistant to at least these 5 drugs. On the other hand, mutations in the *pncA* gene conferring pyrazinamide resistance were diverse, indicating individual and more recent drug resistance acquisition under selective pressure (e.g., failing treatment regimens) (Figure 4). Similar patterns can be observed for the other 2 outbreaks: shared first-line resistance mediating mutations; unique mutations mediating resistance to kanamycin (*eis* −8 del c, −10 g/a, −12 c/t, −14 c/t, −15 c/g, −35 g/a, −37 g/t); cross-resistance to all injectable drugs (*rrs* 1401 a/g); and resistance to the fluoroquinolones (*gyrA* A90V, S91P, D94G, D94N, D94Y, D94A) (Figure 4; Appendix 1 Table 1). We did not identify any known resistance marker for cycloserine and linezolid (Appendix 1 Table 2).

Strains of the 3 outbreak clades acquired putative compensatory mutations, e.g., in *rpoB* or *rpoC* previously suggested to mitigate the growth deficit of rifampin-resistant strains (*10**,**11*). These mutations occur either jointly or after the rifampin resistance mediating mutation in the *rpoB* rifampin resistance determining region (Figure 4).

## Discussion

This study demonstrates that MDR/XDR TB patients in southern Ukraine are infected predominantly with strains belonging to 3 distinct MTBC Beijing outbreak clades. The fact that strains belonging to 2 of these outbreaks have been isolated from patients elsewhere in the world indicates long-term transmission and prevalence of these strain types on a broader geographic scale (i.e., Eastern Europe and Central Asia). Each outbreak subgroup harbored specific combinations of mutations mediating drug resistance, a likely result of clonal expansion of a drug-resistant common ancestor giving rise to almost identical clones that individually acquired additional drug resistance mediating mutations.

Considering the mean pairwise distances among the outbreak clades (14 SNPs for Ukraine outbreak, 25 for Central Asia outbreak, and 39 for Europe/Russia outbreak) and a proposed short-term mutation rate for MTBC strains of ≈0.5 SNPs/genome/year (*23*), we estimate that each outbreak progenitor emerged 20–40 years ago. The W148 Europe/Russia outbreak clade and the Central Asia outbreak clade have been described previously in former Soviet Union territories (*8**,**10*). The Ukraine outbreak clade has not been found in other settings and may be geographically restricted to Ukraine.

Imprisonment has been discussed as the major driver of MDR/XDR TB transmission in Ukraine (*28**–**30*). However, only 1 of the Ukraine outbreak case-patients had been previously imprisoned. The contact tracing review indicates household/neighborhood and nosocomial transmission as modes for the spread of MDR/XDR outbreak strains. The fact that 25 of 53 outbreak strains (Appendix 1 Table 1) have been isolated from new TB cases in our study suggests larger transmission networks in the community. However, because of limitations of the contact tracing review, it is possible that we overlooked some contacts connected to prisons in Ukraine.

The resistance profiles of the outbreak strains indicate that TB treatment regimens including isoniazid, rifampin, pyrazinamide, ethambutol, kanamycin, amikacin, capreomycin, levofloxacin, moxifloxacin, ethionamide, prothionamide, para-aminosalicylic acid, linezolid, and cycloserine (*31*) are not sufficient to effectively treat patients infected with these strains. Indeed, the implementation of rapid Xpert MTB/RIF diagnostics (Cepheid) in the Odessa region has not resulted in substantially improved MDR TB treatment outcomes (*32*). The failing standard MDR TB regimens that include 2–3 active and 2–3 nonactive drugs are still used instead of personalized, laboratory-confirmed treatment (*30*). One reason for this is a lack of the universal access to new or repurposed anti-TB drugs such as bedaquiline, delamanid, and meropenem, and limited funding of palliative TB care that should be used when fewer than 4 active drugs are available (*31*).

Studies in South Africa and Argentina demonstrate that inadequate treatment regimens are drivers for clonal expansion of particular strain types and further drug resistance acquisition (*13**,**33*), which also holds true for the expansion of the 3 detected Beijing outbreak clades in southern Ukraine. In fact, the observed mutation profiles of the outbreak strains with identical first-line drug resistance–mediating mutations and individual mutations that confer second-line drug resistances are characteristic of an acquired MDR MTBC infection (primary resistance), followed by further resistance development under suboptimal treatment regimens (secondary resistance) during a more recent infection or treatment regimen. The high prevalence of high-level isoniazid resistance, mediated by *katG* S315T, and also the broad spectrum of pyrazinamide resistance mediating mutations adds Ukraine to the list of countries where the recently proposed standard short MDR TB regimen will have hardly any effect (*34**,**35*).

The main limitation of our study is that only 21.81% of all MDR isolates registered during the study period could be recovered within routine practice because of limited technical and human resources. This shortfall could potentially introduce some uncertainties regarding the true proportions of the outbreak clades. Further, we show that MDR/XDR TB patients in southern Ukraine are infected mainly with 3 distinct MTBC Beijing outbreak clades. However, inhabitants of southeastern Ukraine were indeed at higher risk for primary MDR TB (*36**,**37*). This finding might be linked to the circulation of the Beijing outbreak strains we describe, but we cannot extrapolate our results to the whole country which was shown to have an asymmetric distribution of MDR TB (*37*). Finally, the low number of genetically predicted and confirmed transmission events in our study is likely due to the short sampling period.

In conclusion, we demonstrate that distinct MDR outbreak clones in combination with failing regimens have been the main drivers of the MDR/XDR TB epidemic in Ukraine, rather than poor adherence to treatment. Individualized treatment regimens based on laboratory-confirmed resistance profiles are the key to containing MDR/XDR TB in these settings. Access to new drugs such as bedaquiline and delamanid should be supported, but strictly controlled, because resistances are likely to develop (*38*).

Appendix 1 Additional information about multidrug-resistant and extensively drug-resistant *Mycobacterium tuberculosis* Beijing clades, Ukraine.

Appendix 2 Additional analysis of multidrug-resistant and extensively drug-resistant *Mycobacterium tuberculosis* Beijing clades, Ukraine.
