# Supplementary material for: Multidrug- and Extensively Drug-Resistant Mycobacterium tuberculosis Beijing Clades, Ukraine, 2015
Source: Emerg Infect Dis. 2020 Mar;26(3):481–90. doi: 10.3201/eid2603.190525 (PMC7045844; doi:10.3201/eid2603.190525)
Supplement: Appendix 2 — Additional analysis of multidrug-resistant and extensively drug-resistant Mycobacterium tuberculosis Beijing clades, Ukraine. [file 19-0525-Techapp-s2.pdf]

Article DOI: https://doi.org/10.3201/eid2603.190525

Multidrug- and Extensively Drug-Resistant *Mycobacterium tuberculosis* Beijing Clades, Ukraine, 2015

Appendix

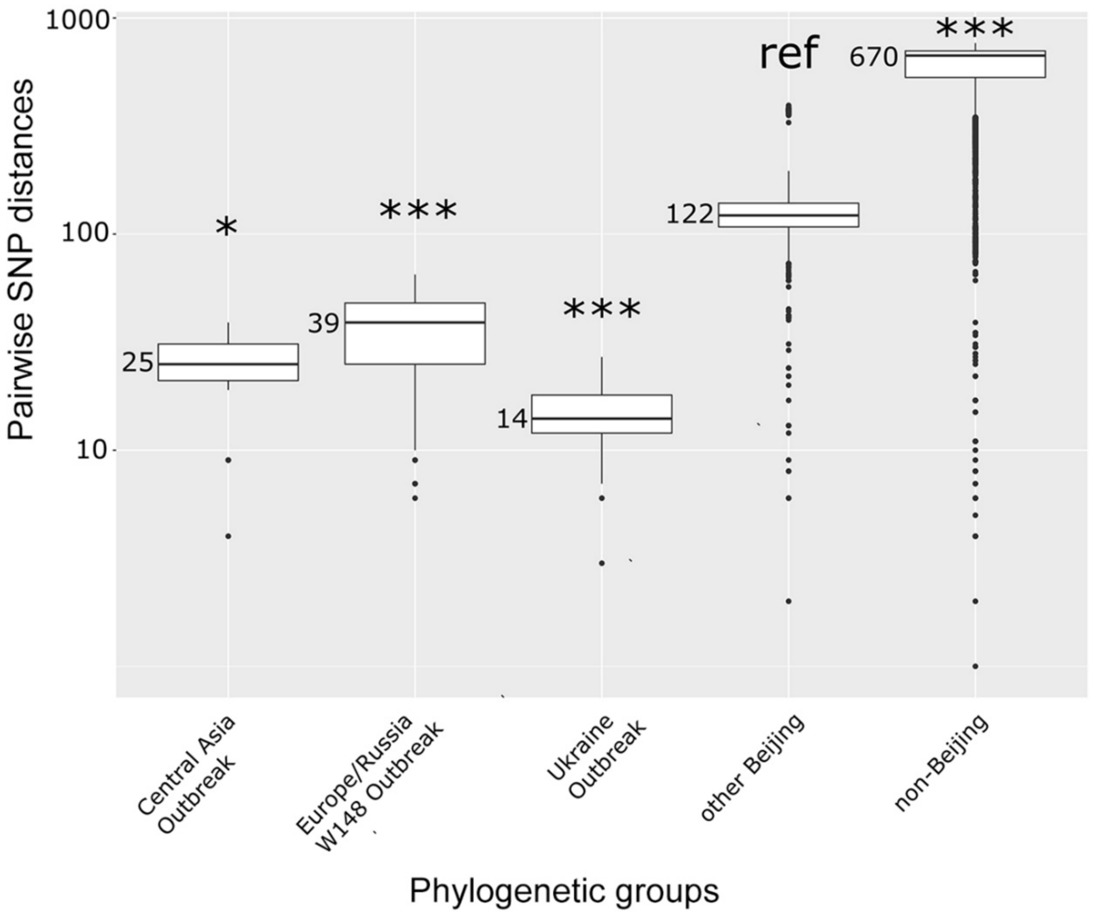

Appendix Figure 1. Box plot showing pairwise single nucleotide polymorphism (SNP) distances among identified Beijing lineage 2.2.1 subgroups in comparison to other Beijing strains and non-Beijing strains. Box represents interquartile range, whiskers represent 95% of the data, black dots represent outliers; solid black line represents the median. *, $p \leq 0.05$, ***, $p \leq 0.001$ compared with reference group (ref) using one-way analysis of variance with Bonferroni multiple comparison test.

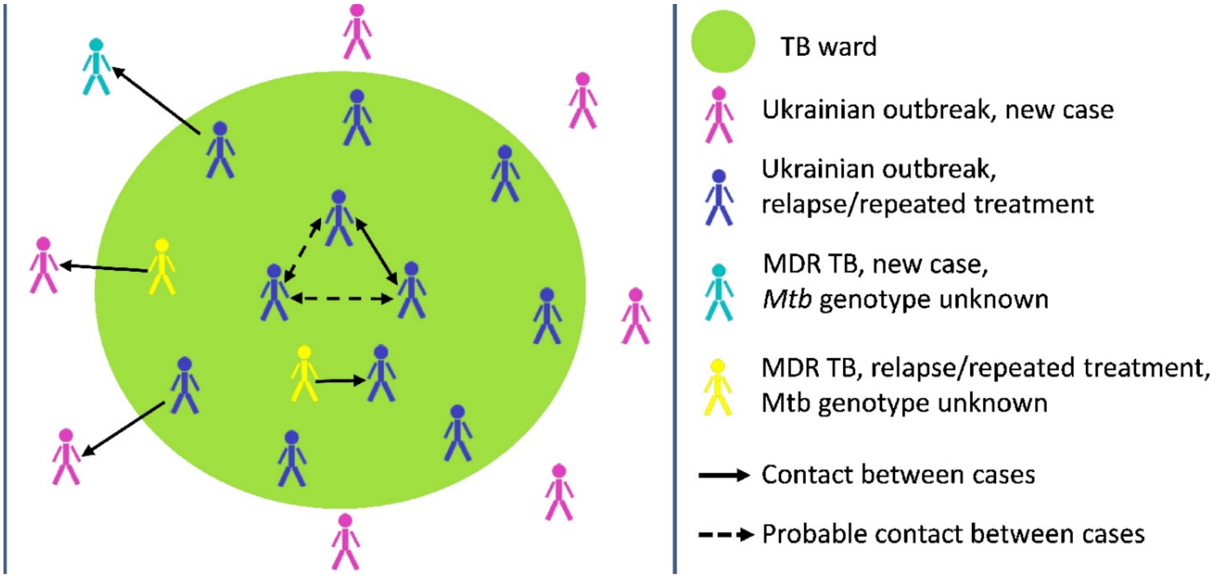

Appendix Figure 2. Contact investigation among patients infected with the Ukraine outbreak strain. All cases located within the green area were treated in a TB ward (inpatient). The direction of an arrow between two cases points from the case that was registered earlier. An arrow points in both directions when the time of MDR TB onset is unclear. MDR TB cases of unknown *Mycobacteria tuberculosis* complex genotype are close family contacts of patients infected with the Ukraine outbreak strain. It is possible that these cases were also infected with the Ukraine outbreak strain but bacterial DNA from these cases was not available.
